# DeSlice: An Architecture for QoE-Aware and Isolated RAN Slicing

**DOI:** 10.3390/s23094351

**Published:** 2023-04-28

**Authors:** Mikhail Liubogoshchev, Dmitry Zudin, Artem Krasilov, Alexander Krotov, Evgeny Khorov

**Affiliations:** 1 Institute for Information Transmission Problems of the Russian Academy of Sciences, 127051 Moscow, Russia; 2 Phystech School of Radio Engineering and Computer Technology, Moscow Institute of Physics and Technology (National Research University), 115184 Moscow, Russia

**Keywords:** network slicing, resource allocation, wireless networks, 5G, network assistance, cross-layer interaction, scheduling, radio access network

## Abstract

Network slicing is considered a key feature of 5G and beyond cellular systems. It opens the door for new business models of mobile operators, enables new services, reduces costs with advanced infrastructure-sharing techniques, and improves heterogeneous traffic service. With slicing, the operators can tailor the network resources to the requirements of specific verticals, applications, and corresponding traffic types. To satisfy the heterogeneous quality of service (QoS) requirements of various slices, efficient virtualization and resource allocation algorithms are required. Such algorithms are especially crucial for the radio access network (RAN) because of the spectrum scarcity. This article develops DeSlice, a novel architecture for RAN slicing. DeSlice enables efficient real-time slicing algorithms that satisfy heterogeneous QoS requirements of the slices and improve the quality of experience for their end users. The article illustrates the advantages of DeSlice by considering the problem of the joint service of cloud VR, video, and web traffic. It develops the algorithms using DeSlice architecture and application-to-network communication. With simulations, it shows that, together, the architecture and the algorithms allow greatly improving the QoE for these traffics significantly.

## 1. Introduction

We witness enormous traffic growth and the emergence of new types of traffic. In addition to well-known web traffic, online gaming, VoIP calls, video conferencing, and IPTV, today’s networks are saturated with dynamic adaptive streaming over HTTP (DASH) (such as YouTube and Netflix) and Internet of Things (IoT) communications. This decade, networks are expected to provide a medium for such demanding applications as industry automation, tactile internet, and augmented and virtual reality (AR/VR) applications. Each traffic type has a specific quality of experience (QoE) model and the corresponding quality of service (QoS) requirements.

5G networks aim to satisfy all these requirements at once. They provide high data rates, high reliability, low latency, high power and spectrum efficiency, high connection, and traffic density [1]. Unfortunately, despite broader frequency bands, the operators’ resources remain limited. Hence, to simultaneously satisfy such heterogeneous and contradictory requirements for complex traffic, 5G networks need to be much more flexible than the preceding generations. Network slicing and virtualization are the primary enablers of such flexibility [2].

Following the network slicing paradigm, multiple virtual networks (slices) can be deployed within a single physical infrastructure. Through network function virtualization (NFV), typical network functions are decoupled from the hardware executing them. Routing, charging, content delivery, and other functions are implemented in software and executed on commodity servers and switches. Similarly, network resources, such as wired links, wireless channels, and antennas, are virtualized and decoupled from the corresponding physical ones.

By now, numerous studies have considered such aspects of network slicing as slice lifecycle management [3], virtual network function placement [4,5,6], slice admission control [7], slice resource management [8], and slice security [9,10].

The essential variability and stochasticity of user behavior, network state, traffic flows, and computational workload require dynamic long- and short-term resource allocation to provide high QoS and QoE and minimize costs for operators. Thus, hypervisors (i.e., slicing algorithms) are required to efficiently multiplex the execution of different virtual network functions on shared servers, data storage in shared databases, and the transmission of data flows over shared wired and wireless links [8]. Computing hardware virtualization and containerization efficiently solves the former issues, and the latter are well studied in the case of wired networks. However, many issues are still open in wireless networks [3].

RAN (radio access network) slicing differs from the core (i.e., wired) network slicing because of the additional stochasticity and variability of the wireless channel and the complex relationship between the available bandwidth and the throughput of different clients [11]. For example, if two clients have different channel conditions (path loss, interference, and fading), they have different channel capacities and consequently need different channel resources to achieve the same throughput. These peculiarities, along with heterogeneous traffic patterns and QoS requirements of different slices, turn the RAN slicing problem into a complex multi-criteria optimization problem. Yet, this problem shall be solved in real time at the base station.

### 1.1. State-of-the-Art RAN Slicing Architectures

The problem of RAN slicing is widely addressed in the literature but still has many unresolved issues [12]. The most important is the trade-off between isolation and efficiency. On the one hand, isolation is one of the central business requirements of mobile operators: a higher degree of isolation allows for revealing less information about the operator’s customers and business models [13]. On the other hand, the higher the degree of isolation, the lower the statistical multiplexing gain and the infrastructure reuse efficiency [14].

**Figure 1:**
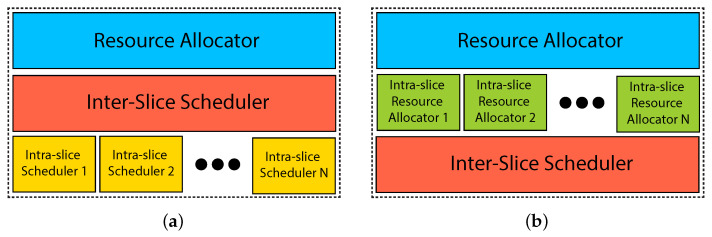
State-of-the-Art RAN Slicing Architectures. (**a**) Bandwidth Isolation used in [15,16]. (**b**) QoS Isolation used in [17,18,19].

The isolation at the bandwidth layer is the most common approach to this trade-off [15,16]. It implies that the infrastructure provider (InP) semi-statically assigns a fixed configuration of channel resources: bandwidth and periodicity. The allocation is done according to the current load and advertised QoS requirements of the slice. The slice operator allocates these resources to its clients according to its policy. If some slices cannot use their resources at some moment, InP redistributes the surplus between the other slices. We can generally illustrate such resource allocation with the RAN slicing architecture shown in Figure 1a.

With this architecture, the resource allocator assigns the amount of channel resource for each slice in the long term. The inter-slice scheduler splits resources between slices in the short term (e.g., per time-transmission interval, TTI) and optimizes the InP-specific utility function under long-term resource allocation constraints. Finally, intra-slice schedulers distribute channel resources assigned to the slice between the slice clients. They are controlled by the tenants and optimize slice-specific utility functions.

Such an architecture provides the desired isolation between the slices and leaves much control to the tenants. However, a limited choice of resources for a slice tenant may significantly degrade statistical multiplexing because of the high time-frequency variability of wireless channel quality. Furthermore, being very limited in resources, a tenant can fail to provide the clients with the required QoS when their channel degrades.

Another approach is the QoS-level isolation [17,18,19] schematically presented in Figure 1b. Here, the tenant creates or modifies the slice with an intra-slice resource allocator, describes the clients’ QoS requirements, and distributes its channel resources between them. Then, the inter-slice scheduler does all the QoS provisioning work and ensures that all clients of different slices are satisfied.

This approach uses the wireless spectrum more efficiently. However, to allocate resources, the InP needs to solve a rather complex multi-objective optimization problem considering the heterogeneous QoS requirements of different slices and the detailed state of the clients of the slices. Therefore, the inter-slice scheduler implemented by the InP shall have complete information about the clients of the slices. As a result, it limits the ability of slice operators to adjust the resource distribution between their clients dynamically. Moreover, the long-term resource allocation of priorities or exact formulation of per-client QoS/QoE metrics move the business models and the intellectual property related to slice management from the tenants to the InP.

### 1.2. Paper Contributions

In this paper, we develop a novel framework to solve the RAN slicing problem while, in contrast to other approaches from the literature, achieving both high spectral efficiency and isolation between the tenants of the slices.

The main contribution of this paper is a novel RAN slicing architecture called DeSlice that (i) helps to satisfy QoS and QoE requirements for heterogeneous flows of different slices; (ii) reduces costs for operators by achieving a higher level of resource utilization and spectral efficiency; (iii) imposes a low computational burden on the network devices; and (iv) provides isolation between the tenants of the slices.

The rest of the paper is organized as follows. In Section 2.1, we present our RAN slicing architecture. Section 4 provides examples of the algorithms using the capabilities of the architecture and the platform, and Section 3 describes their extensive performance evaluation. Finally, we give concluding remarks in Section 5.

## 2. DeSlice Architecture for Improved QoE and Isolation

**Figure 2:**
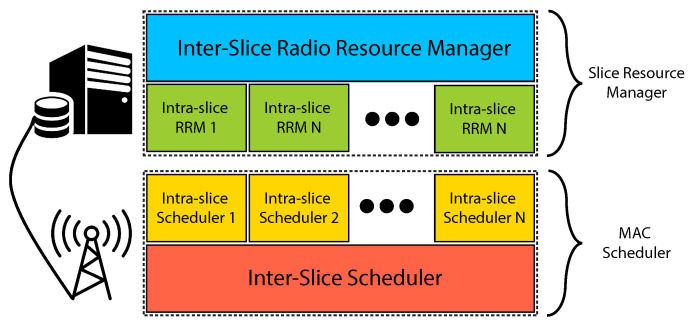
The DeSlice architecture.

In this section, we develop a novel RAN-slicing architecture called DeSlice. The rationale behind its design is as follows. The network slicing problem is a complex multi-criteria optimization problem because various slices and traffic types have different QoE requirements evaluated with different, often non-convex, metrics at timescales of a session. However, the 5G base station, called the next-generation NodeB or gNB, has to solve the resource allocation problem in real time. Hence, to simplify the multi-criteria optimization, we should decompose the problem into a few simpler single-criteria ones. In particular, we decompose the problem into mutually dependent long- and short-term resource allocation problems inside slices and between them [20]. Figure 2 presents the hierarchical structure of the developed architecture.

### 2.1. DeSlice Building Blocks

Let us discuss each layer of the hierarchy in detail.

The slice resource manager (SRM) is a long-term resource allocation instance. It can be installed in the core network as a part of the management and orchestration (MANO) framework. The main objective of SRM is to allocate the shares of channel resources to slices and flows. It makes decisions infrequently, so it can solve complex resource allocation problems and optimize the network performance in the long term.

SRM has two levels. The inter-slice radio resource manager (RRM) allocates the shares of channel resources to the slices and is implemented by the InP. The shares can be assigned by an agreement between the InP and tenants or calculated based on the activity of the clients of slices inside a single cell or in the network in general. The intra-slice RRM allocates the shares of channel resources or assigns the long-term priorities to particular clients of the slice and is implemented by the slice tenant. The basic intra-slice RRM allocates equal priority and a fair share of resources to each of the clients of the slice. However, the DASH-video slice can benefit from centralized video bitrate selection [20,21]. In that case, the tenant allocates shares according to the selected bitrates of the videos downloaded by the clients.

Unlike SRM, MAC Scheduler performs short-term (per-TTI) scheduling. To reduce delays, we implement it at the gNB. The slice tenant implements the intra-slice scheduler or chooses one from the schedulers already implemented by the InP and tailored for the tenant’s type of service. The intra-slice scheduler aims to optimize QoE for the particular traffic type. Traffic-based slice selection for a client allows decomposing heterogeneous QoS requirements. It obtains resource allocation information from the intra-slice RRM, virtualized channel state information from the inter-slice scheduler, and picks the best client for each virtual resource element based on the chosen policy. Finally, each scheduler reports the list of its clients for virtual resource elements sorted by the measure of instant importance of the resource to them.

An inter-slice scheduler is a hypervisor. It abstracts the physical layer and a part of the MAC layer for the other levels of architecture so that for the intra-slice schedulers, the channel appears as a set of virtual resource elements. For example, in the case of single-user transmissions in 5G, these resource elements can be directly mapped to the physical resource blocks. For every client of a slice, each resource element is characterized by its quality (a proxy of achievable channel rate) and a measure of instant importance assigned by the corresponding intra-slice scheduler. Then, the inter-slice scheduler allocates physical resources to the clients based on their importance metrics, QoE requirements, and long-term shares of resources allocated to the slices.

Notice that the inter-slice scheduler does not need to allocate the exact predefined shares of resources to each slice in each TTI. Instead, it shall provide target shares of resources in a time window. Thanks to this, the inter-slice scheduler (i) improves the average spectral efficiency by prioritizing slices and clients experiencing the best channel conditions in particular TTIs and resource elements; and (ii) improves QoE provisioning by prioritizing slices and clients having stringent QoS requirements (e.g., small remaining lifetime and low buffer level).

Using the DeSlice architecture, the tenants can control the service of their clients. At the same time, the InP can take into account the fine-grained channel dynamics and use advanced wireless resource allocation techniques, such as MU-MIMO or CoMP transmissions.

### 2.2. Cross-Layer Interaction

The DeSlice architecture allows us to solve the RAN slicing problem efficiently and improve the QoE of the end users. However, we need to retrieve its requirements and status online to achieve the best performance for each flow. For over-the-top (OTT) services, such as YouTube and Netflix, the requirements are typically available only at the application level. At the same time, the intermediate network nodes are unaware of the nature of the traffic. Such limitations stem from privacy and fairness concerns: users would not like to expose the content they consume to third parties, and OTT service providers would like network neutrality to be preserved. That is possibly one of the reasons why the framework of bearers in LTE networks is underutilized [22].

To realize the benefits of the 5G networks and provide excellent QoE for flows with heterogeneous and often contradictory requirements, we have to sacrifice privacy to some extent. However, we have to admit that it has already been sacrificed: the general architecture of the LTE/5G network includes traffic detection function since Release 12 [23]. This function is typically performed by deep packet inspection (DPI) software or hardware produced by such companies as Qosmos, Allot, Sandvine, and others. Unfortunately, modern DPI systems have limited performance, and the detection accuracy of the most advanced machine-learning-based methods recently considered in the literature is noticeably below 100% [24,25,26]. Therefore, while the privacy of the users is already violated, QoE provisioning still cannot be significantly improved because of DPI performance limitations. That is why establishing direct communication between the applications running on the end nodes and the intermediate network equipment is appealing. If carefully designed, such cross-layer communication would not introduce additional privacy threats, while the provided metadata would help the operators to improve QoE. As a bonus, such communication simplifies the adaptation of the application to the changing network state. For example, instead of indirectly measuring the network capacity and congestion, the end-client applications can ask the network [21].

To date, numerous application-to-network communication protocols or APIs have been developed. Some examples are SAND [27], xStream [21], and xMB [28]. This paper assumes that the network uses xStream because it provides the functionality required to distinguish between different traffic types and exchange QoE-related recommendations.

With xStream, DeSlice can classify traffic and exchange service information about type-specific QoS requirements between UEs and the network. Based on this information, long-term radio resource managers can accurately estimate the amount of resources required for each slice and each slice client. Moreover, for some traffic types, e.g., DASH-video streams, the RRM can help solve the bitrate allocation problem. At the same time, the schedulers in the architecture can obtain and use valuable information about the served traffic in real time. Such information includes the state of the communication channel with the UE, packet deadlines, levels of client video buffer occupancy, and sizes of web pages downloaded by clients.

## 3. Case Study: DeSlice for Cloud VR, Video and Web Traffic Service

### 3.1. Scenario and Problem Statement

To show the benefits of the proposed slicing architecture, we use it to maximize the QoE for clients running web, video, and cloud VR applications. For that, we first need to briefly describe these applications, their QoE models, and the corresponding QoS requirements.

The set of web slice clients is $U^{web}$ download web pages using HTTP. Their QoE depends on the web page download delay. More specifically, according to ITU [29], excellent subjective QoE corresponds to a download delay below two seconds. We assume that $u_{i}\in U^{web}$ is satisfied when the average web page download delay, $\delta_{i}^{web}$, does not exceed two seconds. The following satisfaction indicator for $u_{i}\in U^{web}$ displays this criterion more formally:

(1) $$1_{u_{i}}^{web}=\left\{\begin{array}{cc} 1\; & \;\mathrm{if}\;\delta_{i}^{web}\leq 2s,\\ 0,\; & \;\mathrm{otherwise}.\; \end{array}\right.$$

The set of video slice clients is $U^{video}$. Video clients download DASH videos [30]. With DASH, a video is split into segments with a duration of several seconds each. Each segment is precoded in different qualities (e.g., different resolutions and bitrates) and stored on a server. A client requests segments one by one using HTTP and plays them back to the user. The video quality can be chosen manually or by an adaptation algorithm that estimates the state of the network and playback. The DASH technology does not specify the algorithm itself and is application-specific. We model video QoE with composite satisfaction criteria for $u_{i}\in U^{video}$: (i) the total duration of the video playback $\theta_{i}^{480p}$ in the resolution not less than 480p shall reach at least 85%, (ii) the initial delay of video playback $\delta_{i}^{video}$ shall be less than 5 s, and (iii) the stall duration $\Delta_{i}^{video}$ shall be less than 0.1% of the whole video playback duration $\Theta_{i}$. Once the criteria are satisfied, the aim is to maximize the average bitrate of the video flows. The following expression gives the satisfaction indicator of $u_{i}\in U^{video}$:

(2) $$1_{u_{i}}^{video}=\left\{\begin{array}{cc} 1\; & \;\mathrm{if}\;\frac{\theta_{i}^{480p}}{\Theta_{i}}\geq 0.85\;\mathrm{and}\;\delta_{i}^{video}\leq 5\;s\;\mathrm{and}\;\frac{\Delta_{i}^{video}}{\Theta_{i}}\leq 0.001,\\ 0\; & \;\mathrm{otherwise}.\; \end{array}\right.$$

The rationale behind the choice of the criteria is as follows. First, a video resolution higher than 480p is frequently unnoticeable for the users of smartphones [31]. Second, the QoE of users decays exponentially with the increasing overall stall duration. Hence, it shall be kept very low [32]. Finally, the QoE decays logarithmically with the growth of initial delay [32]. Hence, we can choose a relatively high value that still allows us to detect if some flows are deferred from service.

The set of VR clients is $U^{VR}$. Cloud VR clients run remote interactive gaming, education, or engineering applications. The cloud VR headsets only capture the actions of the users and send them to remote servers. The servers update the virtual reality based on the most recently received actions, encode it into a video sequence and send it to the headsets. The headsets play back the video to the users, perhaps slightly adjusting the displayed picture to the actual position of the users. In such a workflow, the cloud VR traffic is a sequence of video frames with stringent delay budgets: 50 ms for interactive applications [33]. We model a cloud VR QoE with the following satisfaction criteria. A cloud VR session is satisfied if more than 99% of frames arrive within the delay requirement [33]. Hence, the network shall allocate enough resources for the cloud VR slice to satisfy all cloud VR sessions consistently. Let us define for $u_{i}\in U^{VR}$ the number of frames that arrive within the delay requirement as $n_{i}^{VR}$ and the total number of frames as $N_{i}^{VR}$. More formally, the following expression defines the satisfaction indicator of $u_{i}\in U^{VR}$:

(3) $$1_{u_{i}}^{VR}=\left\{\begin{array}{cc} 1\; & \;\mathrm{if}\;\frac{n_{i}^{VR}}{N_{i}^{VR}}\geq 0.99,\\ 0\; & \;\mathrm{otherwise}.\; \end{array}\right.$$

In the paper, we assume that the InP’s revenue grows with the number of satisfied users of each slice. Additionally, the revenue received from a satisfied VR user is much higher than the revenue from a web or video user. Except for VR users, who require specific service with high bandwidth and low delays, the InP preserves the network neutrality and aims to provide a fair resource distribution between the elastic traffic types: web and video. Summing up, we can formulate the RAN slicing problem as the following problem of the revenue maximization of InP:

(4) $$\max\left(\sum_{u_{i}\in U^{web}}1_{u_{i}}^{web}\cdot W_{i}\;+\;\sum_{u_{i}\in U^{video}}1_{u_{i}}^{video}\cdot W_{i}\;\;+\;\sum_{u_{i}\in U^{VR}}1_{u_{i}}^{VR}\cdot W_{i}\right),\;s.t.\frac{S_{video}}{S_{web}}=\frac{N_{video}}{N_{web}},$$

where the revenue of the InP for a satisfied VR flow $W_{VR}\gg W_{web}=W_{video}$, i.e., the revenue of the InP for a satisfied web or video flow.

### 3.2. Proposed Solution

Let us describe the algorithms that operate at each of the layers of the architecture and help us solve the optimization problem.

#### 3.2.1. Inter-Slice Radio Resource Management

The top architecture level is the long-term inter-slice RRM. We consider cloud VR as high-priority traffic because it generates more revenue and it is inelastic and non-adaptive, unlike web and video. In other words, providing it with less-than-required resources is equivalent to no service. Hence, first, the inter-slice RRM allocates enough resources to satisfy the requirements of the cloud VR slice:

(5) $$S_{VR}=\sum_{u_{i}\in U^{VR}}w_{i}^{VR},$$

where $w_{i}^{VR}$ is the share of resources required to satisfy the VR UE *i* defined in the following section. Please note that we do not discuss the problem of admission control in this paper, and we consider the scenarios with $S_{VR}<1$.

Then, the algorithm allocates the remaining resources, namely $1-S_{VR}$, to the web and video slices proportionally to their average number of active clients, i.e., clients that have data to be downloaded:

(6) $$S_{video}=\left(1-S_{VR}\right)\cdot \frac{N_{video}}{N_{video}+N_{web}},\;S_{web}=\left(1-S_{VR}\right)\cdot \frac{N_{web}}{N_{video}+N_{web}}.$$

Such allocation allows us to achieve fair resource distribution between the slices with elastic traffic types.

#### 3.2.2. Intra-Slice Radio Resource Management

The second level is the long-term intra-slice RRM, which differs for each slice.

First, cloud VR sessions require a stable bandwidth that ensures the necessary cloud VR stream bitrate. Therefore, the long-term resource share of a cloud VR client is calculated as follows:

(7) $$w_{i}^{VR}=\frac{b_{i}}{C_{i}\left(t\right)},$$

where $C_{i}\left(t\right)$ is the average channel capacity, and $b_{i}$ is the target bitrate of the cloud VR stream of UE *i*.

Second, for video clients, we implement centralized video bitrate selection that helps avoid the mistakes of the client-side adaptation that can happen due to the fluctuations in the network service rate, following [34]. Specifically, initially, each user is assigned the minimal acceptable bitrate of 480p. Then while some resources in the video slice are still idle, the bitrate of video users is gradually incremented. Thus, each flow gets a long-term share of resources that shall be enough to deliver the video with the bitrate corresponding to the selected resolution:

(8) $$w_{i}^{video}=\frac{b_{i}}{C_{i}\left(t\right)},$$

where $b_{i}$ is the bitrate of the video of UE *i* assigned by the bitrate selection algorithm.

Finally, all web flows have the same priority and shall receive the same long-term resource shares. For that, we distribute evenly the share allocated to the web slice between all active web flows:

(9) $$w_{i}^{web}=\frac{S_{web}}{N_{web}\left(t\right)}.$$

#### 3.2.3. Intra-Slice Scheduling

For each slice, we select the schedulers that optimize the service of the corresponding traffic type.

Namely, we select the shortest remaining processing time (SRPT) [35] algorithm as a web slice scheduler because it aims at minimizing the average duration of a web page download. In OFDMA resource block *k*, we select the UE $\hat{i}$:

(10) $$\hat{i}=\arg\max_{i\in U_{web}}\left(\mu_{ik}^{SRPT}\left(t\right)\right)=\arg\max_{i\in U_{web}}\left(\frac{r_{ik}\left(t\right)}{S_{i}\left(t\right)}\right),$$

where $r_{ik}\left(t\right)$ is the amount of data that can be transmitted to UE *i* in slot *t* in resource block *k*, and $S_{i}\left(t\right)$ is the amount of data remaining to be transmitted to UE *i* before the completion of web page loading.

Next, the SAND-enabled bitrate and resource allocation (SEBRA) algorithm [34] prioritizes video clients with low video buffers, reducing the probability of video stalls. In particular, in resource block *k*, we select the UE $\hat{i}$:

(11) $$\hat{i}=\arg\max_{i\in U_{video}}(\mu_{ik}^{SEBRA}\left(t\right))=\arg\max_{i\in U_{video}}\left(\frac{r_{ik}\left(t\right)}{\bar{r_{i}}\left(t\right)}e^{T_{i}\left(t\right)/Q_{i}}\right),$$

where $T_{i}\left(t\right)$ is a token, which tracks the difference between the allocated throughput and the target bitrate of the UE, $Q_{i}$ is the video buffer level, and $\bar{r_{i}}\left(t\right)$ is the average throughput of UE *i*.

Finally, the MLWDF scheduler [36] serves cloud VR slice because it prioritizes the packets with the most stringent delay requirements and gives them the best resources:

(12) $$\hat{i}=\arg\max_{i\in U_{VR}}(\mu_{ik}^{MLWDF}\left(t\right))=\arg\max_{i\in U_{VR}}\left(\frac{D_{i}\left(t\right)r_{ik}\left(t\right)}{C_{i}\left(t\right)}\right),$$

where $D_{i}\left(t\right)$ is the age of the head-of-line packet for UE *i*.

#### 3.2.4. Inter-Slice Scheduling: Constant Channel

Finally, due to the high complexity of the original optimization problem, we develop two different heuristics for the inter-slice scheduling algorithms at the lowest level of the DeSlice architecture: one assuming that the wireless channel is constant and another that additionally benefits from the channel fluctuations.

For the first slicing algorithm, we consider only slice priority for scheduling: while the higher-priority queue is not empty, any lower-priority one receives no service. Cloud VR traffic has very stringent QoS requirements, and therefore is served first. If there are cloud VR data to transmit, we allocate all the resources to the cloud VR slice. Next, to optimize the download rate of the web pages, we should postpone video service as long as possible. Thanks to the application-to-network communication, we know the buffer levels of the video clients and the sizes of their video segments that are yet to be downloaded. Therefore, we can schedule the video segment transmissions to finish just before the client’s buffer becomes empty and allocate the remaining resources to the web slice. The long-term resource allocation by the RRM guarantees that cloud VR and video flows do not consume all the resources, and the fair share will remain available for web traffic.

Such a scheme allows us to satisfy the requirements of cloud VR flows and improve the QoE of the web and adaptive video flows. However, it leaves large room for optimization because the state of the wireless channel varies in both time and frequency domains. Taking this into account, we can additionally improve the performance of the system with channel-aware slicing.

#### 3.2.5. Inter-Slice Scheduling: Dynamic Channel

The concept of the channel-aware inter-slice scheduler is based on the weighted proportional fair scheduler [37]. This scheduler maximizes the weighted sum of logarithms of the average throughputs of the clients. We modify the original algorithm to optimize the throughput of different clients at configurable timescales. For that, we change the optimization problem by assigning different smoothing factors (effectively averaging windows) to clients generating traffic with different QoS requirements.

Consequently, we aim to solve the following optimization problem. Let $s_{i}\left(t\right)$ be the amount of data transmitted to the UE *i* in TTI *t*. Then, $\bar{r_{i}\left(t\right)}=\left(1-\frac{1}{\tau }\right)\bar{r_{i}\left(t-1\right)}+\left(\frac{1}{\tau }\right)\frac{s_{i}\left(t\right)}{TTI}$ is the exponentially weighted moving average of the throughput in the time window τ:

(13) $$U=\sum_{i}w_{i}\cdot log\left(\bar{r_{i}\left(t\right)}\right),$$

where $w_{i}$ equals the target long-term share of resources allocated for UE *i* by the corresponding intra-slice RRM algorithms.

The modification of the utility function changes its gradient and, therefore, the scheduling metric of the algorithm. Nevertheless, the optimization problem remains convex. Applying the gradient descent algorithm to the problem, in RB *k*, in TTI *t*, we select for scheduling the UE $\hat{i}$:

(14) $$\hat{i}=\arg\max_{i}\left(\mu_{ik}^{WPF}\left(t\right)\right)=\arg\max_{i}\left(\frac{w_{i}}{\tau }\cdot \frac{r_{ik}\left(t\right)}{\bar{r_{i}\left(t\right)}}\right).$$

The moving average window defines the time interval at which the QoS requirements of a slice are satisfied. In other words, it depends on the timescale at which the slice is insensitive to isolation violation. Hence, we can choose the averaging windows of the clients according to their QoS requirements as follows. For cloud VR, the time interval corresponds to its frame-delay requirements, i.e., from tens to hundreds of milliseconds. For DASH video, it is in the order of the video buffer size, i.e., dozens of seconds. Finally, the window for web traffic has the order of the web page download time, namely, a few seconds.

**Figure 3:**
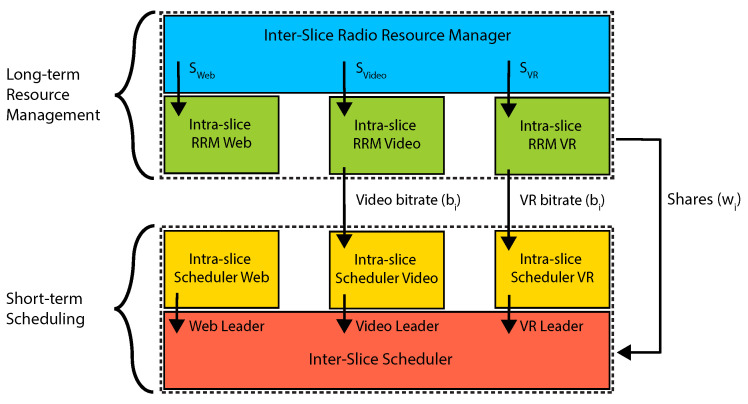
Interaction between the building blocks of the DeSlice architecture.

To summarize the proposed solution, the pseudocode of the short-term scheduling algorithms is presented in Algorithm 1 and the interaction between the blocks of DeSlice architecture is shown in Figure 3.
**Algorithm** **1:** Inter-Slice Scheduling
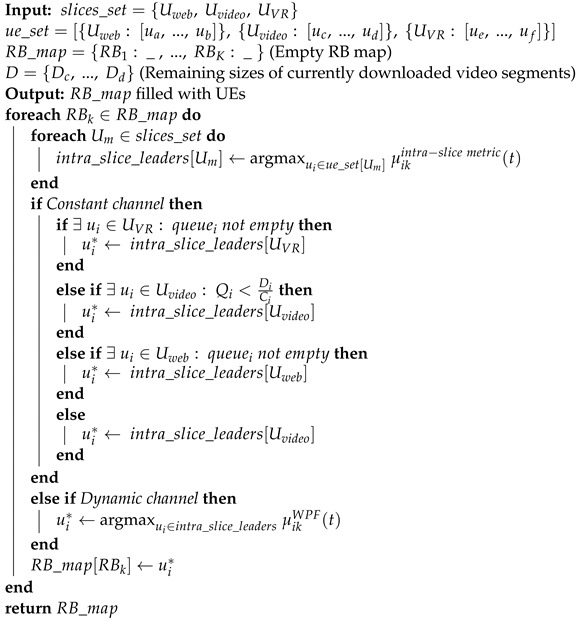


## 4. Numerical Results

We use a widely popular NS-3 [38] simulation platform to evaluate the performance of the DeSlice architecture with different inter-slice scheduling algorithms. We consider a single-cell network, where *N* UEs are randomly dropped around the gNB in a circle of radius R=500 m. The gNB has wired connections to a cloud VR server in the InP network and the web and video servers on the Internet.

The UEs generate either web, video, or cloud VR traffic. Web UEs download pages with sizes drawn from the empirical distribution retrieved from the HTTP archive database [39]. They request web pages with a truncated exponentially distributed inter-request time with the mean of 45 s, minimum of 15 s, and maximum of 90 s. Video UEs use DASH to download videos picked from the database of popular YouTube clips [40]. Their operation points correspond to the resolutions: {144p,…, 360p,…, 1080p}. Cloud VR video stream is a trace generated by a state-of-the-art cloud VR Pico Neo Streaming Assistant application [41] with an average bitrate of 15 Mbps and 60 frames per second frame rate. Each video and VR session has the uniformly distributed duration of [90,110] s, and the duration of the inter-session interval of each UE has a truncated exponential distribution with the mean of 30 s, the minimum of 10 s and the maximum of 60 s. Table 1 shows the other simulation parameters.

**Figure 4:**
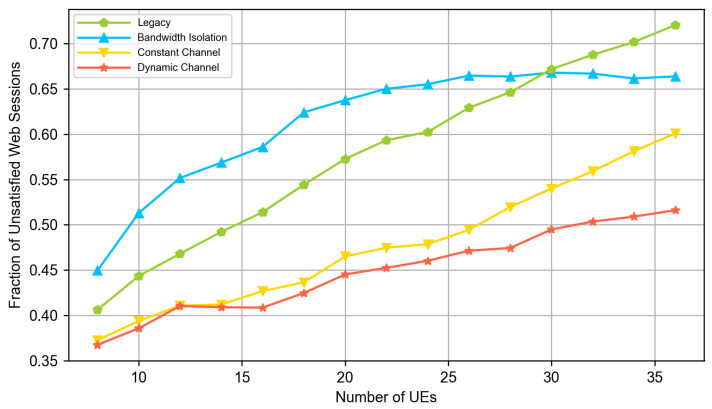
The fraction of unsatisfied web sessions.

**Figure 5:**
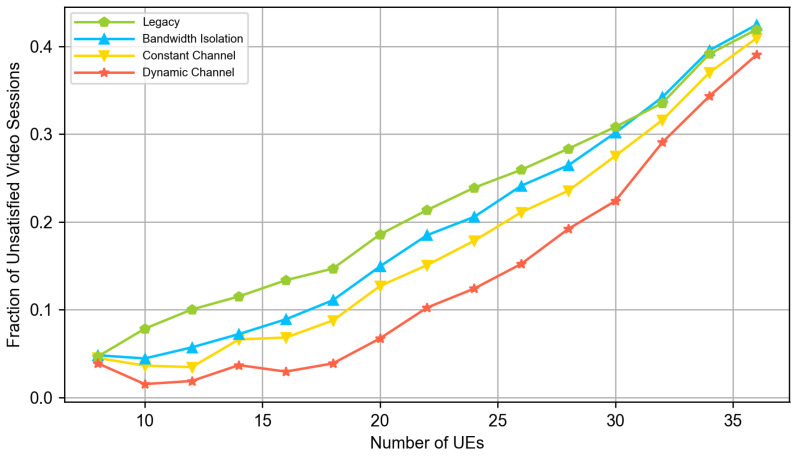
The fraction of unsatisfied video sessions.

**Figure 6:**
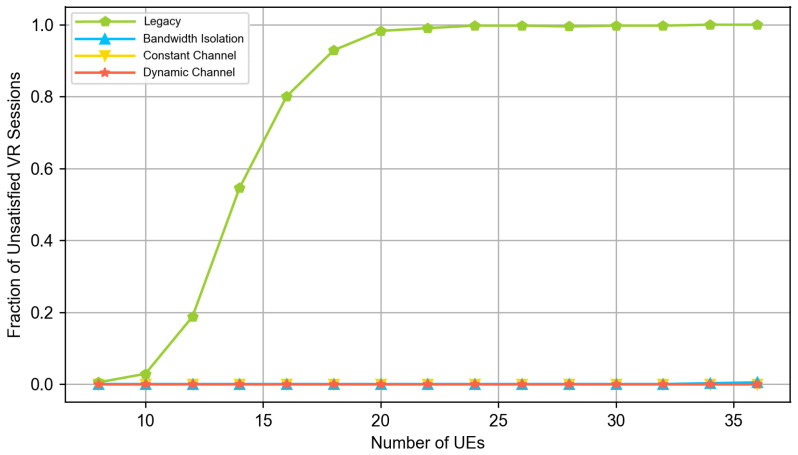
The fraction of unsatisfied cloud VR sessions.

In our experiments, there are always two cloud VR UEs, half of the remaining UEs are video clients, and another half are web clients. We vary the total numbers of UEs in the system and evaluate the QoE of the end users using the QoE models defined in Section 3.1. Figure 4, Figure 5 and Figure 6 compare the QoE provided by the following solutions:

- *Legacy (no slicing):* the default 5G system and DASH video application.
- *Bandwidth isolation:* RAN slicing architecture considered in the papers [15,16]. To provide a fair comparison and solve the problem described in Section 3.1, we extend the NVS slicing algorithm described in [16] with the same allocation of long term shares of resources as in our solution, i.e., the shares are allocated as described in Section 3.2.1 and Section 3.2.2.
- *Constant channel:* DeSlice architecture with solutions described in Section 3.2 and constant channel inter-slice scheduler described in Section 3.2.4.
- *Dynamic channel:* DeSlice architecture with solutions described in Section 3.2 and dynamic channel inter-slice scheduler described in Section 3.2.5.

Figure 5 and Figure 6 show that at low system loads, the network has enough resources to satisfy almost all video and cloud VR sessions when using any solution. However, Figure 4 shows that the DeSlice-based solutions can improve QoE upon the legacy system and bandwidth-isolating slicing for web flows by prioritizing them when the video and cloud VR traffic does not require immediate service. Note that the observed fraction of unsatisfied web sessions is well above zero because the 2 s threshold for the web page download time corresponds to excellent service, which cannot be achieved for edge users when the base station serves two cloud VR flows.

Figure 6 shows that the legacy system fails to satisfy cloud VR traffic requirements starting from the medium loads. The main reason is that the cloud VR slice requires more resources than the fair share provided by the legacy system. Meanwhile, DeSlice-based solutions provide higher QoE for web and video traffic types and keep all cloud VR sessions satisfied (see Figure 4 and Figure 5). Overall, the developed solutions reduce the fraction of unsatisfied web sessions by up to 40% and reduce the fraction of unsatisfied video sessions by up to 2.5 times.

Finally, at high loads, we outline the following. First, Figure 5 shows that the DeSlice-based solutions gradually reduce video QoE and bring it close to the QoE of the legacy system. It happens because the considered inter-slice RRM focuses a lot on the cloud VR service and allocates its slice more-than-fair share of resources. Hence, the video slice gets fewer resources in slicing-enabled systems than in the legacy system, and clients watch videos with an unsatisfactory resolution (less than 480p). Nevertheless, compared to the legacy system, slicing-enabled solutions still reduce the fraction of unsatisfied web sessions by up to 30% and satisfy 100% cloud VR sessions at the same time (see Figure 4 and Figure 5). Second, the *dynamic channel* solution provides higher web and video traffic QoE than the *static channel* and *bandwidth-isolating* solutions. In the considered scenario, it reduces the fraction of unsatisfied web sessions by up to 30% and the fraction of unsatisfied video sessions by up to 20% compared to the *static channel* solution. Finally, compared to a *bandwidth-isolating* solution, the *dynamic channel* solution reduces both the fraction of unsatisfied web and video sessions by up to 25%. The high relative performance of the *dynamic channel* solution illustrates why the inter-slice scheduler shall consider both the channel state of the clients of the slice and their QoS requirements.

## 5. Conclusions

In the paper, we presented DeSlice, a novel RAN slicing architecture that enables flexible and efficient resource allocation between and within slices. The architecture helps to decompose a complex resource allocation problem into a few simpler mutually dependent optimization problems and solve them in real time. Furthermore, the proposed architecture allows tenants to control the service of their clients and provides high resource utilization, high spectral efficiency, and resource isolation at flexible timescales.

We demonstrated the advantages of DeSlice architecture by solving a RAN slicing problem for joint web, video, and cloud VR traffic services. With simulations, we showed that the proposed solution significantly improves QoE. In particular, the developed solution achieved up to 50 and 35% gain in web and video QoE over the RAN slicing architecture and algorithms known from the literature. Unlike legacy systems, it satisfied the requirements of 100% cloud VR flows.

## Figures and Tables

**Table 1:** Simulation parameters.

Parameter	Value
Channel	20 MHz @ 2 GHz
Channel model	3GPP TR 38.901 EPA
gNB/UE TX power	30/23 dBm
gNB antenna type	Omni-directional
gNB height	30 m
UE height	1 m
TTI duration	1 ms
Wired connection capacity	10 Gbps
Duration of simulation run	1000 s
Number of simulation runs	20

## Data Availability

The implementation of the developed algorithms can be found here: http://wireless.iitp.ru/network-slicing/, accessed on 16 April 2023.

## Abbreviations

The following abbreviations are used in this manuscript:

AR/VR	Augmented and Virtual Reality
DASH	Dynamic Adaptive Streaming over HTTP
gNB	Next-Generation NodeB
InP	Infrastructure Provider
IoT	Internet of Things
MANO	Management and Orchestration
MAC	Media Access Control
NFV	Network Function Virtualization
OTT	Over-the-Top
QoE	Quality of Experience
QoS	Quality of Service
RAN	Radio Access Network
RRM	Radio Resource Manager
SRM	Slice Resource Manager
TTI	Time-Transmission Interval
UE	User Equipment
